# The Scientific Value of Abstracts on Prostate Cancer Presented at the European Association of Urology Congresses

**DOI:** 10.3389/fsurg.2021.683359

**Published:** 2021-06-15

**Authors:** Jasmin Ataei, Christian Bach, Aida Javan, Thomas-Alexander Vögeli, Christina Grafe, Mohammad Sajjad Rahnama'i

**Affiliations:** Department of Urology, Uniklinik Rheinisch-Westfälische Technische Hochschule (RWTH) Aachen, Aachen, Germany

**Keywords:** abstracts, conferences, prostate cancer, publication, congress

## Abstract

**Background:** Scientific congresses are an important medium for presenting recent clinical findings. Publication of abstracts allows wider dissemination.

**Objectives:** To determine the publication rates of prostate cancer abstracts presented at the annual congress of the European Association of Urology (EAU).

**Design, Setting, and Participants:** All abstracts with the term prostate cancer or carcinoma presented at the congress of the European Association of Urology from 2015 to 2018 were analyzed. We captured their publication rate, journal impact factor and time to publication. Moreover, we formulated a scoring system to determine the grade of discrepancy between the conclusions mentioned in the congress abstract and published abstract.

**Results:** A total of 834 abstracts presented at EAU annual meeting included prostate cancer or carcinoma in their title. We recorded a publication rate of 56.8% with 474 of the 834 abstracts being published with a mean time of 12.5 months.

**Conclusion:** Approximately, 57% of the prostate cancer abstracts presented at the EAU congress are published in peer reviewed journals. This acceptance rate indicates the high distribution and dissemination of these abstracts.

## Introduction

The aim of scientific meetings is to provide a platform where scientists can debate and present research results (1). It brings investigators from different countries together and enables scientific exchange. A part of congress abstracts are approved “as an oral presentation, a printed poster, or an electronic poster.” (2).

Every year hundreds of research abstracts are presented at the European Association of Urology (EAU) Congress, which is the largest urological event in Europe (3, 4). It is a great occasion for urologists to introduce their latest findings to the urologic community. Before these abstracts appear on the EAU congress they have to go through a peer review process.

As the number of medical congresses is increasing, their scientific quality becomes an important issue (5). One way to describe the quality of the scientific content of a congress is to calculate the publication rate of studies following presentation at the meeting (5).

Publication is an important step to disseminate research wider and to increase the chance to reach a larger audience and eventually reach the patient (6). Therefore, it takes a crucial part in the medical development (7). Particularly prostate cancer, which is the most frequent diagnosed cancer in men, is an extensive subject field at urologic scientific meetings (8, 9). Consequently rapid dissemination of clinical findings through full text publication could lead to progression in diagnosis and therapy of prostate cancer and improve the patient managment.

According to a Cochrane review, combining research from 425 reports in different medical fields, only 37% of abstracts presented at congresses are published in peer reviewed journals (10).

The aim of our study is to determine the publication rates of abstracts on prostate cancer (PCa) presented at the EAU congress. In addition, we analyzed the discrepancy between abstract and the corresponding published full paper.

## Materials and Methods

All abstracts with the term prostate cancer or -carcinoma presented at the annual EAU congresses between 2015 and 2018 were included in our study. We used the European Urology Supplements (EUS) of march of each year to gather all abstracts with those keywords in their title.

**Figure 1:**
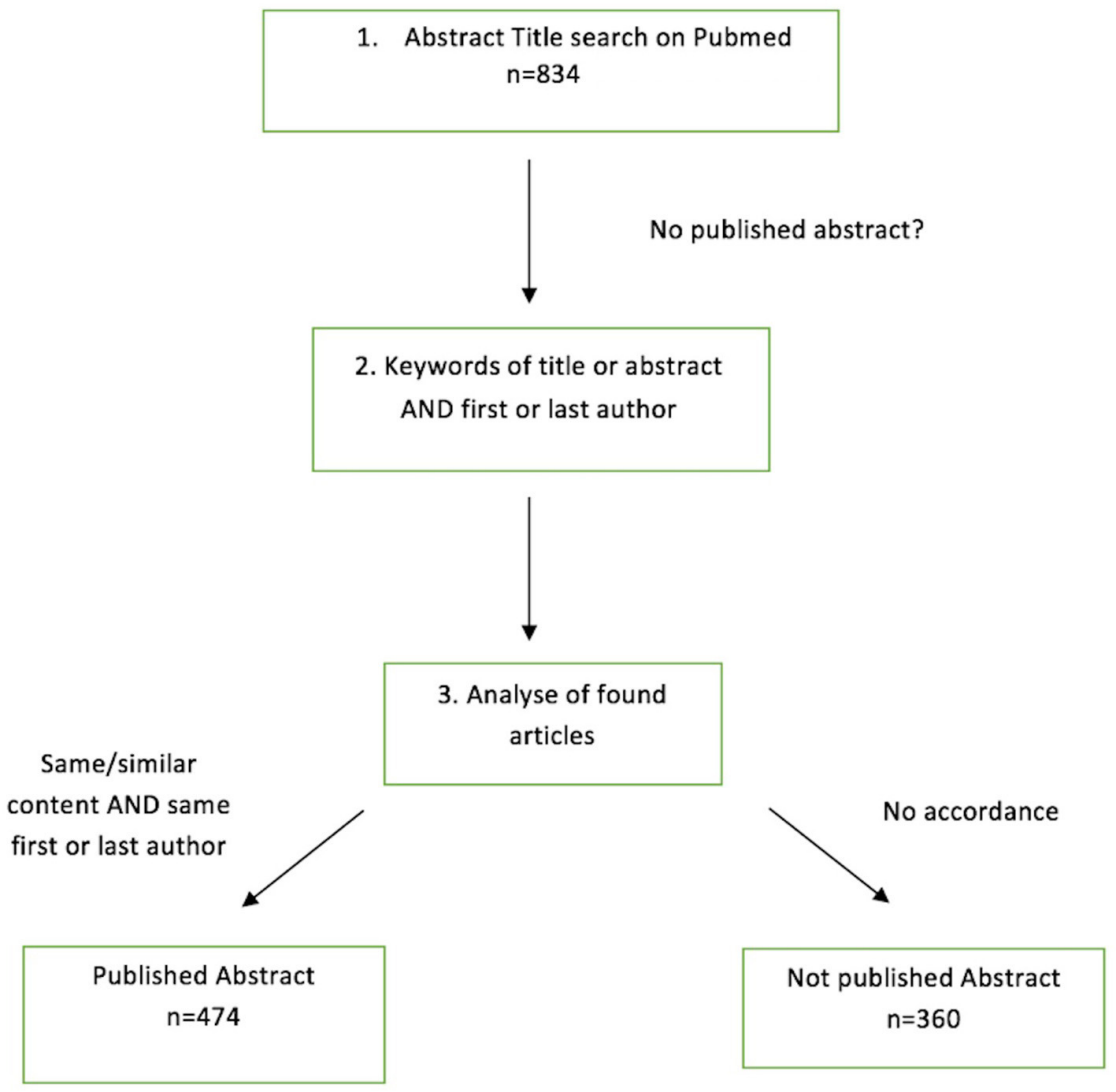
Search criteria.

In a second step the complete abstract title was searched on Pubmed. If no corresponding publication was found, keywords of the title of the abstract and the first or last author were searched. The abstract was scored as published if the congress abstract and the publication had the same or similar content and the same first or last author (Figure 1).

If the congress abstract was published as a full article, the year of publication (electronic publication), the title of the journal and its impacts factor were recorded. The interval between congress and publication dates were captured in full months. The mean time included all abstacts which were published after the congress.

We analyzed and compared the congress abstract with the paper abstract, with the center of attention on the aim of the study, methods, results and conclusion. For this, we defined a scoring system to determine the grade of discrepancy between the congress abstract and the published abstract.

We distinguished between four grades of discrepancy: Grade 0 is defined if the congress abstract and the published abstracts contain the same data, same content, same conclusion or one different data point but still the same content and same conclusion. Grade 1 is determined if we have two or more changed data points but the same content and same conclusion or same data points and one further content, but the same conclusion. Data discrepancy is assigned to Grade 2 if the data is similar with maximal one further content and same or adapted conclusion. If the paper contains further investigation based on the abstracts research, more data, more content and same or different conclusion, we choose Grade 3. Different data points include changes in number of patients, methods, material or results.

## Results

In total, 5,128 abstracts were presented at the EAU Congress of 2015 to 2018. Of these, 834 abstracts included the term prostate cancer or prostate carcinoma in their title.

**Figure 2:**
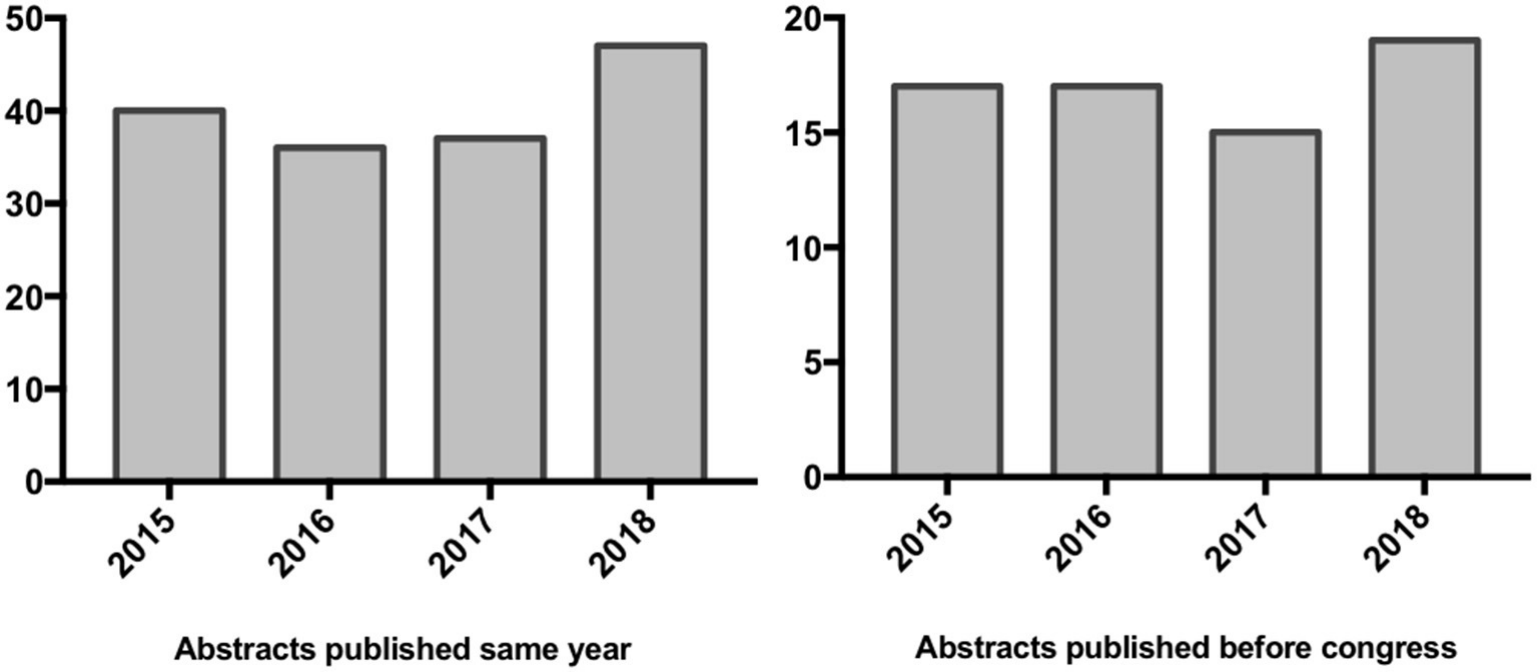
Publication rates of abstracts published same year and before congress.

Of these 834 abstracts 57% were published as full paper in peer reviewed journals (Table 1). The overall mean time to publication was 12.5 months and the overall mean impact factor was 6.236. We scored a discrepancy grade of 1.4 (Table 2). A total of 193 of the 834 (23%) abstracts were published within the same year as the congress. About 10% were published before the annual congress (Figure 2).

**Table 1:** Publication rates of presented EAU congress abstracts and Mean time to publication.

	**2015**	**2016**	**2017**	**2018**	**Total**
Abstracts on PCa	211	191	192	240	834
Abstracts on PCa published	136 (64%)	115 (60%)	99 (52%)	124 (51%)	474 (57%)
Published same year	55 (40%)	41 (36%)	39 (36%)	58 (47%)	193 (23%)
Published before congress	23 (17%)	19 (17%)	15 (15 %)	24 (19%)	81 (10%)
Mean time to Publication	14	14	13	9	12.5

**Table 2:** Impact factor and arithmetic mean of the discrepancy grades.

**Congress year**	**Impact factor**	**Discrepancy grades**
2015	5.910	1.4
2016	5.646	1.3
2017	6.0399	1.5
2018	7.078	1.1
Total	6.236	1.3

**Figure 3:**
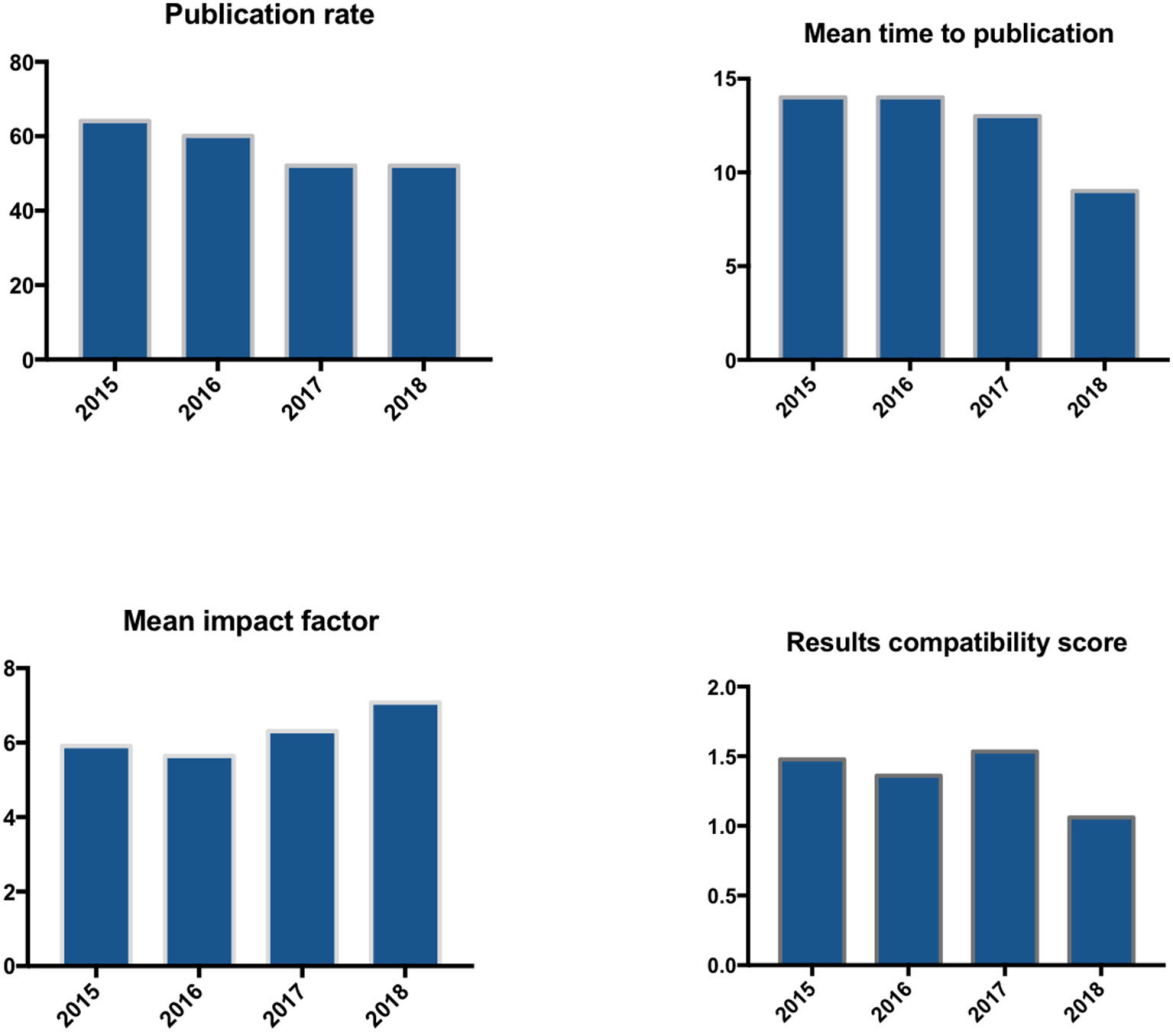
Publication rate (PR), mean time to publication, impact factor and discrepancy score from 2015 to 2018.

Of all prostate cancer abstracts presented at the EAU congress in 2015, 64% were published in full paper, 60% of those in 2016, 52% of those in 2017, and 52% from 2018 (Figure 3).

The mean impact factor increased over the years, whereas the mean time to publication decreased (Figure 3).

These abstracts were published in 146 different journals, with the majority appearing on the European Urology Journal (11%), BJU International Journal (9%) and on the World Journal of Urology (6%) (Table 3).

**Table 3:** Journals publishing EAU congress presentations.

	**Title of Journal**	**EAU- PCa Abstracts published (*n*)**	**%**	**Impact factor (2018)**
1	European Urology	54	11	17.298
2	BJU International Journal	44	9	4.524
3	World Journal of Urology	27	6	2.761
4	The Journal of Urology	24	5	5.647
5	The Prostate	21	4	2.876
6	European Urology Focus	16	3	
7	Prostate Cancer and Prost. Diseases	13	3	4.600
8	Clinical Genitourinary Cancer	10	2	2.450
9	Oncotarget	9	2	
10	International Journal of Clinical Oncology	8	2	2.503

We found 527 poster presentation and 307 oral presentations. Fifty-eight percentage of these published abstracts were poster presentations whereas 54% were oral presentations (Table 4).

**Table 4:** Oral vs. poster presentations.

	**Poster presentations published *n* (%)**	**Oral presentations published *n* (%)**
2015	88 (64%)	48 (66%)
2016	76 (61%)	39 (56%)
2017	53 (52%)	46 (51%)
2018	91 (56%)	78 (33%)
Total	308 (58%)	166 (54%)

In 2015, 64% abstracts were published, 40% were published in the same year as the congress and 17% before it. We have a mean time to publication of 14 months and a mean impact factor of 5.9. The discrepancy grade is 1.4.

In 2016, 60% were published in peer reviewed journals, 36% were published in the same year as the congress and 17% before it. We captured a mean time to publication of 14 months and a mean impact factor of 5.6. The discrepancy grade is 1.3.

In 2017, 53% abstracts were published, 36% were published in the same year and 15% before the congress. We have a mean time to publication of 13 months and a mean impact factor of 6.04. The discrepancy grade is 1.5.

In 2018, 52% were published in peer reviewed journals, 47% were published in the same year as the congress and 19% before it. We have a mean time to publication of 9 months and a mean impact factor of 7.08. The discrepancy grade is 1.1. The discrepancy grades of each years were listed in Table 5.

**Table 5:** Discrepancy grades of each years.

	**2015**	**2016**	**2017**	**2018**	**Total**
Grade 0	39	31	17	39	126
Grade 1	24	35	38	49	151
Grade 2	47	33	26	23	127
Grade 3	26	15	18	13	69

## Discussion

We analyzed the publication rate of 834 abstracts presented at the annual EAU congress. Fifty-seven percentage of PCa abstracts presented at the EAU from 2015 to 2018 are published in full paper and can be found on Pubmed. This acceptance rate seems to be higher compared to other urological events. Lower rates of 33.9% were reported by Rao et al. for the British Association of Urological Surgeons Annual Meeting (11). Autorino et al. analyzed abstracts presented at the EAU congress from 2000 to 2001 with a publication rate of 47.3% (4). In addition, a publication rate of 47.2% of the abstracts presented at the European Society for Pediatric Urology meetings from 2003 to 2010 has been reported (12).

Comparable results of 55% publication rates were found in 2006 for the American Urology Association annual meeting (13). The mean time between congress and publication (12.5 months) is shorter compared to other medical meetings (5, 14).

The publication rate decreased over the last years. One of the main reasons for this decrease could be the shorter follow up time from the actual congress. This also could explain the decrease of the mean time to the publication. The mean Impact Factor has increased over the last years. This can mainly be explained by the fact that the impact factor of the European Journal, which included most of the papers, has increased over the recent years. Nevertheless, our mean impact factor of 6.236 is high and reflects the high standard of the abstracts presented at the congress.

The acceptance rate of poster presentation was slightly higher than the oral presentation rate (58 vs. 54%).

The annual EAU meeting thematized the most important fields of prostate cancer such as diagnosis, therapy and prognosis. It enables urologist from all of Europe to communicate and exchange their findings. In order to spread their clinical work internationally, their aim should be publication in peer reviewed journals (7). But there are still numerous reasons why abstracts are not published, either because of rejection by the submitted journal or the fact that the authors never try to present their work for publication to a journal (2).

Scherer et al. investigated the reasons for unpublished research and found that “lack of time” and “lack of resources” are the main factors (15). It is known that it takes more work and time to prepare a manuscript than an abstract (9, 16). Therefore, many authors show low motivation and participation and don't attempt to publish their work (17).

A study by Udovicich et al. found out that abstracts which include multicentre studies and bigger groups had a greater chance to get published (18). Furthermore, it is shown that positive outcomes are associated with a higher acceptance rate (19–21). In addition, Bonfield et al. observed that beside factors like positive findings “oral presentations,” “randomized controlled trials” and “smaller meetings” lead to higher conversions rates (21).

In our study, we additionally analyzed the discrepancy grade between the congress abstract and published abstract with our own developed scoring system (Table 6). The arithmetic mean of the discrepancy grade decreased from 2015 to 2018. It turned out that the score was quite low which reflects the accurate reproduction of the abstracts presented at the congress. Consequently, urologists can rely on the scientific investigations presented at the EAU congress.

**Table 6:** Definition discrepancy grades.

**Grades**	**Definition**
0	Exactly the same data and conclusion OR one different data point, but same content and conclusion
1	Two or more changed data points but same content, same conclusion OR same data points and one further content, but same conclusion
2	Data similar, maximum one further content, adapted conclusion
3	Further investigation based on the abstracts research, more data, more content, same or different conclusion

Limitations of our study include that we only searched in Pubmed and only analyzed the EAU congress. Furthermore, we could have a longer follow up time but it is shown in numerous studies that most of the congress abstracts are accepted within the first 2 years of the annual congress (4, 9, 22). Moreover, it is possible that we have missed published abstracts, but we reduced the risk by following our search algorithm, in which we repeat the authors name and the keywords in several steps. Another point to mention is that we did not investigate the type of study and study design, which may also affect the publication rates (10).

## Conclusion

Our study shows that more than half of the PCa abstracts of the EAU are eventually published as full articles high quality peer reviewed journals and indexed on Pubmed. This finding emphasizes the high standard of the work submitted to the annual EAU congress and the sound review process. Our scoring data support these results as content showed to be consistent. Nevertheless, a significant amount of abstracts are not published and consequently these abstracts are withheld from a larger audience.

## Data Availability Statement

The raw data supporting the conclusions of this article will be made available by the authors, without undue reservation.

## Author Contributions

JA, CB, CG, and MR has made substantial contributions to conception and design. JA, AJ, and CB were involved in acquisition of data. JA has been involved in drafting the manuscript and have made substantial contributions to analysis and interpretation of data and to revise it critically for important intellectual content. JA and CB made substantial contributions to statistical analysis. All authors contributed to the article and approved the submitted version.

## Conflict of Interest

The authors declare that the research was conducted in the absence of any commercial or financial relationships that could be construed as a potential conflict of interest.
